# Characterizing and Modulating Brain Circuitry through Transcranial Magnetic Stimulation Combined with Electroencephalography

**DOI:** 10.3389/fncir.2016.00073

**Published:** 2016-09-22

**Authors:** Faranak Farzan, Marine Vernet, Mouhsin M. D. Shafi, Alexander Rotenberg, Zafiris J. Daskalakis, Alvaro Pascual-Leone

**Affiliations:** ^1^Temerty Centre for Therapeutic Brain Intervention, Centre for Addiction and Mental Health, University of TorontoToronto, ON, Canada; ^2^Berenson-Allen Center for Non-invasive Brain Stimulation, Beth Israel Deaconess Medical Center, Harvard Medical SchoolBoston, MA, USA; ^3^Neuromodulation Program, Department of Neurology, Boston Children's Hospital, Harvard Medical SchoolBoston, MA, USA

**Keywords:** transcranial magnetic stimulation, electroencephalography (EEG), TMS-EEG, biomarker discovery, signal processing, experiment design, brain mapping, neuromodulation

## Abstract

The concurrent combination of transcranial magnetic stimulation (TMS) with electroencephalography (TMS-EEG) is a powerful technology for characterizing and modulating brain networks across developmental, behavioral, and disease states. Given the global initiatives in mapping the human brain, recognition of the utility of this technique is growing across neuroscience disciplines. Importantly, TMS-EEG offers translational biomarkers that can be applied in health and disease, across the lifespan, and in humans and animals, bridging the gap between animal models and human studies. However, to utilize the full potential of TMS-EEG methodology, standardization of TMS-EEG study protocols is needed. In this article, we review the principles of TMS-EEG methodology, factors impacting TMS-EEG outcome measures, and the techniques for preventing and correcting artifacts in TMS-EEG data. To promote the standardization of this technique, we provide comprehensive guides for designing TMS-EEG studies and conducting TMS-EEG experiments. We conclude by reviewing the application of TMS-EEG in basic, cognitive and clinical neurosciences, and evaluate the potential of this emerging technology in brain research.

## Introduction

The combination of transcranial magnetic stimulation (TMS) with electroencephalography (EEG) offers an *in-vivo* method for investigating the function and integrity of brain circuits during various behavioral states across the human lifespan. *In-vivo* investigations can provide insights into the mechanism of action of TMS in probing and modulating neural processes, and fundamental new knowledge about distributed brain activity. In parallel, the same methodology can be applied in animal studies or *in*-*vitro* for pre-clinical and mechanistic assessments, and can offer valuable translational phenotypes. In this article, we describe and review TMS-EEG methodology, data acquisition and processing with the intent of introducing the technique to novice users, and offering a comprehensive review for experienced practitioners. We provide an overview of the TMS-EEG mechanism of action, equipment specifications, recording guidelines, and outcome measures. We review parameters that must be carefully chosen when designing TMS-EEG experiments, and provide a big-picture system diagram to guide TMS-EEG study designs. We then in details describe factors that impact TMS-EEG outcome measures including stimulation parameters, tissue properties, brain state, and recording artifacts. We review the challenges in preventing and correcting TMS-EEG artifacts, list existing and potential solutions, and provide a system diagram for conducting TMS-EEG experiments. We conclude by reviewing TMS-EEG applications, drawing examples from previous experiments in basic science, cognitive neuroscience, and clinical research, and briefly comment on the future technical advancements and application of this emerging technology in brain research.

## The working principle of TMS-EEG

### Transcranial magnetic stimulation

#### Mechanism

**Figure 1:**
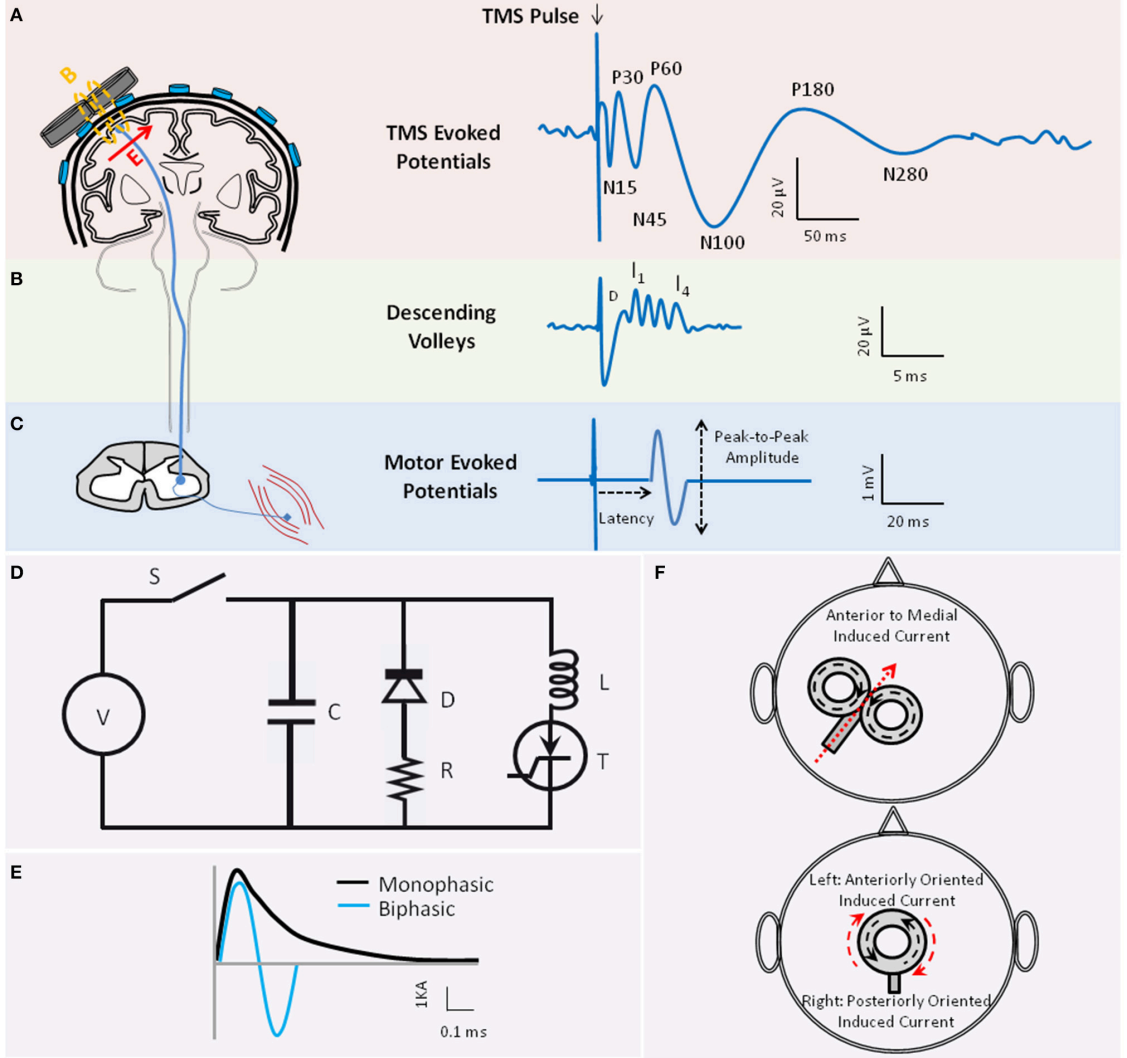
**The basic principle of transcranial magnetic stimulation**. Figure depicts the schematics of TMS-evoked potentials (TEP)s **(A)**, TMS-induced descend volleys **(B)**, and TMS-induced motor-evoked potential (MEP) **(C)** when TMS is applied to the motor cortex. **(A)** The waveform illustrates the average TEPs recorded through electroencephalography (EEG) from a hypothetical EEG sensor close to the vertex. When applied to the motor cortex, several negative (N) and positive (P) TEP components are reported at different latencies (in ms) relative to the time of TMS delivery, including the N15, P30, N45, P60, N100, P180, and N280 (reviewed in Komssi and Kahkonen, 2006). Several of these peaks are associated with activation of specific excitatory and inhibitory neural circuitries. The TMS coil schematic also depicts that the TMS-induced magnetic field (B) is perpendicular to the plane of the TMS coil. The induced electric field in the tissue (E) is in turn perpendicular to the magnetic field. The direction of the induced current in the tissue is anti-parallel to the direction of the current in the coil [E arrow going into the page or coming out]. **(B)** Waveform illustrates the schematic of the TMS-induced descending volleys that can be recorded from the spinal cord, and the direct (D) and indirect (I) waves that are induced in the corticospinal tract depending on the TMS pulse intensity and coil orientation. The D and I waves are associated with direct or transsynaptic activation of pyramidal neurons, respectively. **(C)** The waveform depicts an MEP recorded from a peripheral muscle through electromyography (EMG). The latency and the peak-to-peak amplitude of the MEP are conventionally employed to examine the integrity of the corticospinal tract. **(D)** Figure depicts the simplified system diagram of a TMS coil (L) attached to a TMS device main unit. The TMS main unit often consists of a Voltage (V) source, Switch (S), Capacitor (C), Diode (D), Resistor (R), and Thyristor (T). **(E)** Waveforms are schematics of monophasic (black) vs. biphasic (blue) TMS pulse shapes, here illustrated by the current (Ampere) in the coil. **(F)** Figures demonstrate two popular coil shapes, the figure-of-eight (top) and the circular (bottom) coil shape. Figures also illustrate the relationship between the direction of current in the coil (black dotted arrows) and the direction of the current induced in the brain tissue (red arrow) which is anti-parallel to the coil current. Please note that for the circular coil placed on the vertex that has anti-clockwise coil current, the induced current in the tissue would be clockwise. Therefore, current in the tissue would be anteriorly oriented on the left hemisphere and posteriorly oriented on the right hemisphere.

TMS was introduced in 1985 (Barker et al., 1985) as a neurophysiological tool to study the integrity of corticospinal pathways in humans. When applied within the guidelines (Rossi et al., 2009; Rossini et al., 2015), TMS provides a non-invasive means to trigger or modulate neural activity. TMS is safe if appropriate precautions are taken and set guidelines are followed (Rossi et al., 2009). In TMS, time-varying currents are generated in an induction coil often held tangential on the scalp over area of interest (Figure 1). Following Faraday's law of electrical current induction, the resulting time-varying magnetic field generates a secondary electric current in nearby conductors, including brain structures parallel to the coil orientation. The induction of secondary currents depends not only on the characteristics of the TMS pulse and the coil, but on several other factors, discussed later. The TMS-induced current either causes direct depolarization of neural structures generating action potentials, or modifies the state of tissue excitability (Kobayashi and Pascual-Leone, 2003).

Application of a single TMS pulse to the motor cortex can generate a compound muscle action potential in a target muscle at the periphery, referred to as the motor-evoked potential (MEP; Figure 1C). TMS-induced MEP is often characterized by its amplitude and latency relative to TMS onset, reflecting integrity of corticospinal tract. Epidural recordings in patients with implanted cervical electrodes (e.g., Nakamura et al., 1996; Di Lazzaro et al., 1999) showed that TMS can generate volleys of descending direct wave (D-wave) and indirect waves (I-waves; Figure 1B). The D-wave is suggested to reflect the direct activation of pyramidal neurons, and I-waves the indirect and transsynaptic activation of pyramidal neurons via depolarized interneurons (Day et al., 1987). Depending on the magnitude and orientation of the induced current in the motor cortex, TMS could predominantly induce I- or D-waves (Di Lazzaro et al., 1998). Similar effects on neural structures are thought to occur when TMS targets other brain regions. Several TMS protocols are designed to investigate neural processes including excitation, inhibition, plasticity or connectivity in the sensorimotor and non-motor systems by applying one or more pulses of specific intensity and frequency to one or more brain regions (see Section TMS Parameters).

#### Equipment

A TMS device comprises the TMS coil (the inductor L) connected to the stimulator main unit. This main unit consists of the voltage source generating the magnetic field, a bank of energy-storing elements (the capacitors C) to generate pulses, the internal resistors (R) associated with the cables, and thyristor switches to switch large currents over a short period of time (Figure 1D). The main unit has a pulse-shaping circuitry that controls the resonant frequency of this RLC circuit and determines the pulse shape. The majority of TMS devices provide monophasic or biphasic pulse shapes of pre-determined pulse width (Figure 1E; Wagner et al., 2007). Utility of insulate-gated bipolar transistors in TMS circuitry is proposed to permit controlling the TMS pulse width within the same device (Peterchev et al., 2008). The depth and pattern of the induced electrical field varies across TMS coil shapes (Figure 1F). The most commonly-used coil shapes are circular or figure-of-eight coils. A variety of coil designs are developed to induce complex electric field (Deng et al., 2014).

### Electroencephalography

#### Mechanism

Human EEG was introduced in the 1920s by Berger (1929). The method enables the non-invasive assessment of neural activity resulting from local and long-range neural communication across different spatial scales at *millisecond temporal resolution* (Ingber and Nunez, 2011). In EEG, the electrocellular activities of tightly-packed neurons, aligned perpendicular and radial to the scalp, propagate to the surface of the scalp while passing through several layers: neural tissue, blood, cerebro-spinal fluid (CSF), dura, skull, and skin. The speed of signal propagation through neural pathways is estimated at 6–9 m/s. Neural activity is conducted through the brain volume to the scalp without any measurable time delay, an effect termed *volume conduction*. When one or more large neuronal populations operate in synchrony, for instance in response to an event, a relatively strong electric field is generated and can be recorded at the scalp. However, the resulting electrical field is still several orders of magnitude smaller than the field induced by TMS. A pair of EEG sensors on the scalp measures the potential differences between two regions. EEG signals generally represent the electrical activities generated through spatial and temporal summation of the excitatory and inhibitory postsynaptic potentials. These EEG-recorded oscillatory activities are thought to arise from a combination of factors: the intrinsic properties of neurons (e.g., the time constant of the voltage-gated channels), structural properties (e.g., propagation speed), the functional properties of neurotransmitter systems, and the network interactions and feedback loops (e.g., thalamocortical, corticocortical, cerebellocortical pathways).

#### Equipment

A modern EEG system consists of multiple sensors (electrodes), amplifiers, and an analog-to-digital convertor for data digitization. As EEG measures electrical potential differences, the brain activity recorded at one sensor is always compared to another value. Two types of EEG recordings are generally considered: continuous recording without a temporally-defined external or internal event; or event-related recordings over multiple repeated trials, relative to the presentation or processing of an internal or external event.

### TMS-EEG working principle

In TMS-EEG experiments, the TMS coil is held tangential over the EEG cap and sensors (Figure 1A). The TMS time-varying magnetic field induces an electric field in nearby conductive brain tissue, including the white and gray matter. Since the induced current is parallel to the coil orientation, the tangential orientation ensures induction of current in the underlying tissue. The induced current can generate action potentials directly or indirectly, or modify the brain state. The action potentials in the stimulated area propagate to the interconnected brain regions through short- or long-range cortico-cortical, thalamocortical, or cerebello-cortical pathways. This cascade of events gives rise to the generation or modifications of excitatory and inhibitory postsynaptic potentials whose spatial and temporal summations are recorded by EEG sensors. TMS-induced EEG potentials are referred to as TMS-evoked potentials (TEP)s. When single-pulse TMS is applied to the motor cortex, TEPs have negative and positive potentials at specific latencies relative to the pulse (Figure 1A; reviewed in Komssi and Kahkonen, 2006).

The strong TMS magnetic field also induces unwanted electric field in nearby conductors including the EEG electrodes, skin, nerves, muscles, skull, and CSF. This can generate large-amplitude artifacts in the EEG signal. As discussed later, some of these artifacts are minimized through dedicated TMS-compatible EEG equipment and noise removal techniques. Furthermore, a number of other TMS-related factors can contaminate EEG recordings. For example, the TMS pulse is associated with a loud click and a tapping sensation, leading to generation of auditory-evoked potentials (AEP) and sensory-evoked potentials (SEP), respectively. TMS can also evoke muscle and nerve activations, and eye blinks or movements. Therefore, in addition to dedicated TMS-compatible EEG hardware, a number of control conditions, data recording considerations, and offline noise removal techniques are employed in TMS-EEG studies discussed in later sections.

### TMS-EEG equipment

The combination of TMS and EEG requires the compatibility of TMS and EEG equipment. Essentially all commercially available TMS stimulators can be combined with a TMS-compatible EEG system. However, the TMS coils coating and the cooling systems should be investigated to not induce noise in EEG. There are also commercially available integrated TMS-EEG systems that include optimized amplifiers and electrodes.

A TMS-compatible EEG system differs from a conventional EEG system in that it includes: (1) appropriate technology to avoid amplifier saturation to minimize artifacts during data acquisition, and (2) appropriate electrode types to avoid electrode movement and TMS-induced Ohmic-heating. The advantages of different TMS-compatible amplifiers and electrodes are reviewed in details elsewhere (e.g., Ilmoniemi and Kicic, 2009; Vernet and Thut, 2014) and are briefly described below.

#### TMS-compatible EEG amplifiers

The majority of the early attempts to combine TMS with EEG failed due to saturation of the amplifiers by the large TMS-induced voltage, which usually exceeded the 5 mV voltage limit of most conventional amplifiers (Ives et al., 2006). Although the TMS pulse is < 1 ms, most amplifiers could not recover for up to several seconds after. Initial TMS-EEG experiments tried to minimize this artifact by placing the recording and reference electrodes several centimeters away from the stimulation site (Cracco et al., 1989). However, this configuration limited the number of sensors and was not ideal for recording from the whole head. A more optimal solution was later introduced by Ilmoniemi et al. (1997). In this pin-and-hold setup, the input of the EEG amplifiers is blocked from −50 μs to 2.5 ms post TMS to prevent amplifier saturation. This is done by a combination of strategies, such as reducing the gain of pre-amplifiers, opening the circuits before high-gain amplifiers, and maintaining the voltage constant at different levels of the circuitry during the TMS pulse (Virtanen et al., 1999).

Another solution for recording EEG in the presence of a strong electrical field is using amplifiers with a wide operational range. In these systems, the de-coupling of amplifiers is not necessary as the amplifiers can capture the full shape of the TMS pulse without being damaged or saturated. The combination of a high sampling rate (e.g., >5 kHz), increased analog-to-digital conversion sensitivity (e.g., < 0.5 μV/bit) and a wide operational range (e.g., >5 mV) enables the recording of a wide voltage range with high sensitivity (sub-microvolt to several milli-volts), permitting accurate recording of low-amplitude brain signals shortly after capturing the true shape of the high voltage TMS artifact (Bonato et al., 2006). Moreover, TMS-compatible EEG systems may employ direct current (DC) amplifiers with wide dynamic ranges that do not contain an initial capacitor that can be saturated (Daskalakis et al., 2008).

Yet another solution is to incorporate a preamplifier to limit the rate of voltage change (slew-rate). As the TMS pulse duration is < 1 ms, limiting the rate of change of voltage change enables continuous recording without amplifier saturation (Thut et al., 2003; Ives et al., 2006). It is advantageous to gate the TMS discharge to the EEG acquisition system clock, thereby allowing artifact subtraction techniques to retain electrophysiological signals that occur close in time to the pulse (Thut et al., 2003, 2005).

#### TMS-compatible electrodes

The TMS time-varying magnetic field creates a secondary current (eddy current) in nearby conductors, including the highly-conductive EEG sensors. This may produce repulsive forces that can cause movements and heating of the ring-shaped EEG sensors (Pascual-Leone et al., 1990). The temperature of EEG electrodes was shown to increase as a function of stimulation parameters, including intensity and the number of delivered stimuli, and physical properties of the electrode, including the electrode diameter and conductivity constant (Roth et al., 1992). To minimize artifacts and the potential risk of skin burns, TMS-compatible EEG electrodes should ideally have a small current-loop area. This can be achieved by cutting a section out of the ring electrodes (Roth et al., 1992). A slit in an annulus-shaped electrode is shown to reduce heating and the DC-offset by an order of magnitude (Virtanen et al., 1999). Moreover, the electrode conductivity mass can be reduced by using conductive plastic pellet electrodes coated with a thin layer of silver epoxy (reviewed in Ives et al., 2006).

## Designing TMS-EEG experiments

### TMS-EEG study design

In general, regardless of TMS-EEG application, TMS-EEG experiments may be fitted into two general categories:

#### TMS-EEG to extract markers of brain health

These studies employ TMS to trigger a specific neural circuitry, and the integrity of the circuit is assessed by quantifying the resulting EEG response. In this approach, TMS is considered as an event, similar to a sensory stimulus in classical event-related potential paradigms. Various TMS protocols are employed to trigger specific brain circuitries (see Section TMS Parameters).

#### TMS-EEG to assess or modulate brain-behavior relationship

These studies employ TMS to interfere with (suppress, facilitate, or interrupt) a neural process. The aim is to assess the functional role of the neural process in a cause-effect manner, or to modulate behavior. EEG can capture and quantify the “normal” brain dynamics associated with a behavioral state. TMS is then applied to interfere with the on-going brain dynamics and EEG can capture the effect of the intervention on neural dynamics or behavior.

**Figure 2:**
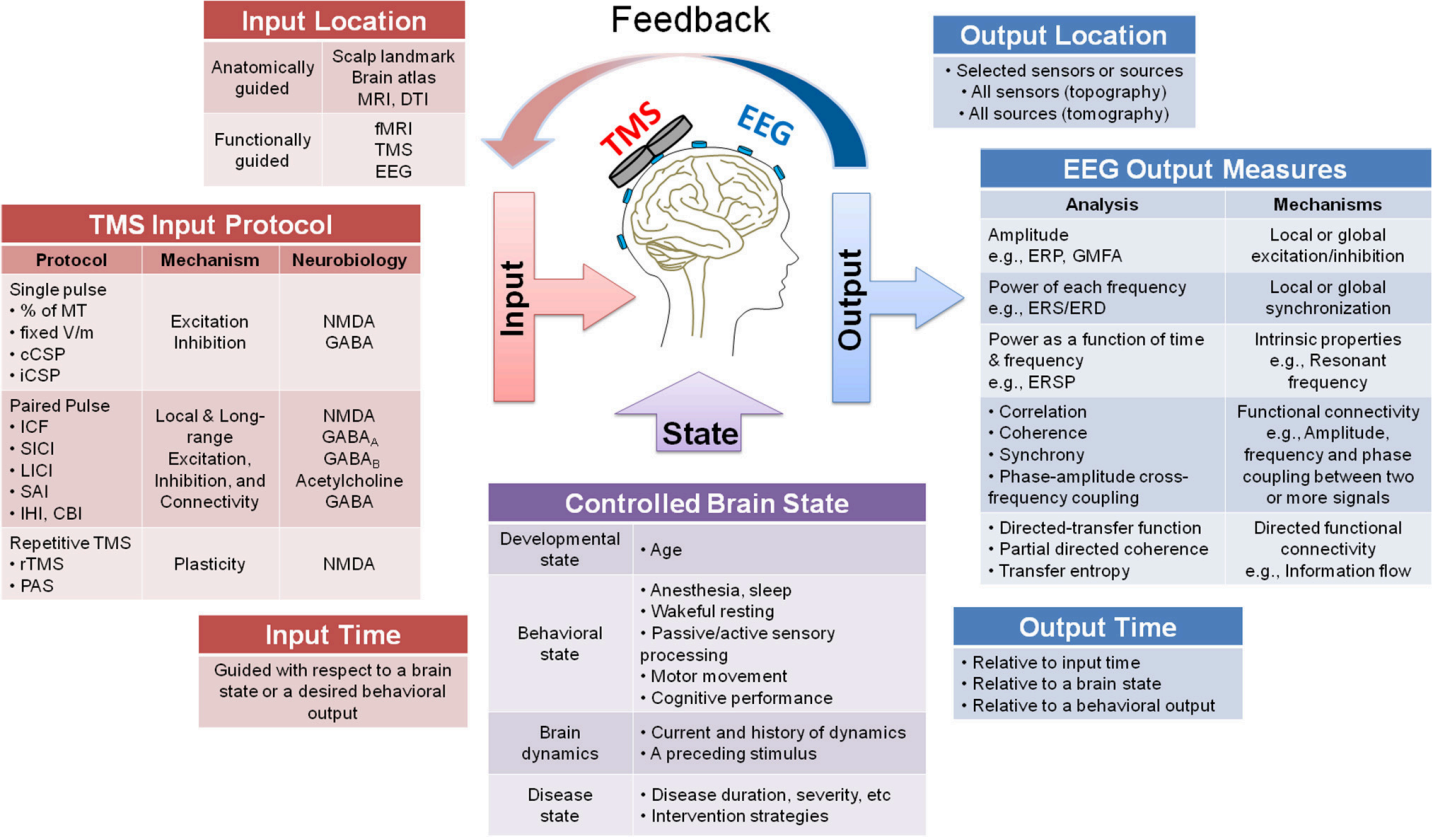
**A system diagram guiding the design of TMS-EEG studies**. The figure presents a system diagram of three main types of parameters that can be selected when designing a TMS-EEG experiment: TMS input parameters (location, protocol, and time), EEG output parameters (location, measures, and time), and brain state parameters (developmental, behavioral, dynamical, and disease states). Several possible choices are listed for each parameter and where applicable mechanism or neurobiology associated with each parameter is specified (e.g., for TMS input protocol and EEG output measures). The figure also depicts the possibility of using the EEG output parameters to guide the TMS input parameters, either offline or through online feedback systems.

The study protocol and parameters should be carefully chosen and controlled for when designing TMS-EEG experiments. Figure 2 presents a system diagram of three types of parameters that can be selected in TMS-EEG experiments: TMS parameters (Figure 2 Input), EEG parameters (Figure 2 Output), and brain state parameters (Figure 2 Brain State). In addition to the listed parameters, several other factors may impact the TMS-EEG outcome measures discussed in subsequent sections.

### TMS parameters

#### Stimulation protocols

In a TMS-EEG experiment, TMS protocols can be employed to target a specific neural circuitry. TMS protocols that are commonly utilized were designed by stimulating the motor cortex and quantifying the impact on the peripheral motor response (Barker et al., 1985), or by quantifying changes in behavior (Pascual-Leone et al., 1996) when non-motor regions are stimulated. Several TMS protocols were designed to investigate excitation, inhibition, plasticity or connectivity in the sensorimotor system. These generally involve quantifying changes in TMS-induced MEP by controlled changes in TMS input parameters (e.g., intensity) and/or application of a preceding conditioning event (e.g., paired-pulse paradigms). Through TMS-EEG, several of these protocols are extended to non-motor regions (Daskalakis et al., 2012) discussed in the Section TMS-EEG Applications.

The most frequently used protocols and metrics include evaluation of motor threshold (MT; Rossini et al., 1994), ipsilateral cortical silent period (iCSP; Wassermann et al., 1991), contralateral cortical silent period (cCSP; Fuhr et al., 1991), and paired-pulse measures such as intracortical facilitation (ICF; Kujirai et al., 1993), short interval intracortical inhibition (SICI; Kujirai et al., 1993), long interval intracortical inhibition (LICI; Valls-Sole et al., 1992), interhemispheric inhibition (IHI; Ferbert et al., 1992), cerebellocortical inhibition (CBI), and short-latency afferent inhibition (SAI; Tokimura et al., 1996). In general, these measures investigate the integrity of a cascade of fast- and slow-acting excitatory and inhibitory processes, occurring either within local cortical circuitry or involving long-range cortico-subcortical feedback loops. Pharmacological studies revealed that each protocol may assess the integrity of specific neurotransmitter systems (reviewed in Ziemann, 2004).

Moreover, neuroplasticity can be assessed by the repetitive application of TMS pulses (rTMS), the repetitive pairing of TMS pulses applied to two brain regions, or the pairing of TMS pulses to a sensory cortex with an appropriately timed peripheral sensory stimulus (paired associative stimulation, PAS; e.g., reviewed in Freitas et al., 2013) which can induce spike-timing dependant plasticity (Stefan et al., 2000). Different rTMS and PAS protocols (e.g., differing in stimulation frequency, pattern, location) can enhance or suppress neural activity beyond the stimulation duration (Pascual-Leone et al., 1994, 1996; Chen et al., 1997). Following active rTMS to the motor cortex, increases or decreases in MEP amplitudes in response to single-pulse TMS of fixed intensity relative to baseline are thought to provide an index of long-term potentiation-like (LTP-like) or long-term depression-like (LTD-like) plasticity (Fitzgerald et al., 2006; Thut and Pascual-Leone, 2010; Vlachos et al., 2012). Plasticity-inducing protocols can have behavioral effects (Pascual-Leone et al., 1996) and might be leveraged for therapeutic applications (Chen et al., 2008).

#### Stimulation location

Numerous methods are used to navigate TMS coil placement. To target the motor cortex, coil position can be adjusted to produce an MEP of maximal peak-to-peak amplitude in a target muscle. Alternatively, coil placement can be guided by *ad-hoc* protocols such as functional or anatomical landmarks [e.g., 5 cm anterior to the motor hot spot for dorsolateral prefrontal cortex (DLPFC)], or by individual's MRI landmark, electrode placement in the EEG 10–20 system (reviewed in Rossini et al., 2015), resting-state or task-based fMRI (e.g., Farzan et al., 2016), or possibly through EEG outcomes. Despite neuro-navigated targeting, the actual brain region that is directly impacted by TMS may vary as a function of several physical and physiological factors, including the TMS stimulation properties (e.g., pulse shape, coil shape, coil orientation), the morphological properties of the stimulated tissue, conductivity index of the stimulated tissue, gyrification of the cortex, and shape and height of EEG electrodes (see Section Factors Affecting TMS-EEG Outcome Measures).

Each method of TMS coil placement has advantages and limitations. EEG 10–20 system is inexpensive but does not account for variability of cortical anatomy across individuals (Rossini et al., 2015). MRI-guided neuronavigation requires obtaining an MRI image for each subject prior to TMS-EEG which is expensive and may not be feasible. Furthermore, coordinates used for identifying functional landmarks (e.g., DLPFC) may be based on group average coordinates and not take into account individual differences. Using individual's task-based or resting-state fMRI coordinates may lead to placement of coil on different scalp locations across subjects. Combined with unevenness of EEG electrode placement, this may introduce other sources of variability due to differences in head shape and scalp-cortex distance, and tissue morphology across subjects. Furthermore, it is possible that functional coordinates may vary over time and as a function of brain state. Thus, unless fMRI is obtained concurrent with TMS-EEG, the coordinates may change between the fMRI and TMS-EEG visits.

#### Implementation of TMS protocols during EEG recording

The extension of TMS protocols, originally defined through quantification of EMG responses, to TMS-EEG experiments requires careful adjustment of several parameters. These include intensity, location, number of stimuli, and brain state.

##### Intensity

The stimulus-response curves of several TMS protocols were documented for EMG outcome measures in the motor cortex (e.g., Sanger et al., 2001; Rossini et al., 2015). Prior TMS-EMG studies have shown the dependency of TMS protocols on proper adjustment of stimulation intensity. For example, the LICI protocol, as defined in the motor cortex at rest, involves two suprathreshold TMS pulses applied 50–200 ms apart, suggested to probe activation of GABA_B_ receptor-mediated cortical inhibition (Valls-Sole et al., 1992). When applied to the motor cortex, the suprathreshold intensity is determined based on the EMG outcome measures often set to 110–120% of resting MT (RMT) or an intensity that on average produces MEP amplitudes of 0.5–1 mV peak-to-peak. Another example is SICI protocol which involves application of subthreshold conditioning stimulus followed by a suprathreshold test stimulus separated by 2–6 ms at rest, suggested to probe activation of GABA_A_ receptor-mediated cortical inhibition (Kujirai et al., 1993). When applied to the motor cortex, SICI conditioning stimulus is set to 80% of RMT and the test stimulus to 110–120% of RMT. Previous studies have documented the impact of modifying the intensity in TMS protocols (e.g., Sanger et al., 2001; Rossini et al., 2015). For example, increasing the intensity of test stimulus may reduce LICI but increase SICI (Sanger et al., 2001).

Therefore, stimulation intensity should be carefully adjusted in TMS-EEG experiments. However, adjustment of intensity in TMS-EEG experiments requires several special considerations. First, EEG electrodes introduce a distance between the TMS coil and scalp. Therefore, the final stimulation intensity should be determined once the EEG cap is placed on the scalp. As discussed in Section Controlling for TMS Click, a thin layer of foam may be placed between the EEG electrodes and the TMS coil to minimize AEP (auditory-evoked potentials) due to bone conduction of TMS click. If this approach is used, the foam should be placed during determination of intensity. The intensity in the motor cortex can then be determined using EMG outcome measures (e.g., RMT) following the set guidelines (Rossini et al., 2015).

When TMS protocols are extended to non-motor regions, however, determination of intensity is not trivial. In such cases, at least three approaches may be used to determine intensity: (1) using the motor cortex threshold determined by EMG outcome measures (e.g., percentage of RMT); (2) using real-time brain-navigated stimulation and adjusting the stimulation intensity by estimation of induced electric field in the brain areas of interest. For example, by determining the induced electric field (V/m) equivalent to 80% RMT or 120% RMT in motor cortex; and (3) utilizing EEG outcome measures.

In the first approach, due to unevenness of EEG electrode layout, the coil-scalp distances may vary across the EEG cap introducing variability across brain regions and subjects. Furthermore, several factors such as head shape, coil orientation, tissue morphology, and scalp-cortex distance may vary across brain regions that could modify the location, distribution and strength of induced electric field (see Section Factors Affecting TMS-EEG Outcome Measures). Moreover, while the stimulus-response curves of TMS protocols are documented for motor cortex using EMG responses, it is possible that these curves may have different characteristic shapes and slopes for non-motor regions. Finally, it is possible that the motor cortex suprathreshold intensity may not activate non-motor tissue with the same strength as illustrated for motor and prefrontal cortices (e.g., Kahkonen et al., 2004; Farzan et al., 2009; Rosanova et al., 2009). Therefore, more investigations are required to capture the stimulus-response curve of TMS protocols using EEG outcome measures in non-motor regions. The brain-navigated approach accounts for several of these shortcomings. However, this approach may not be available in all commercially available neuro-navigation systems or accessible to investigators. Moreover, the accuracy of real-time estimation of the induced electric field depends on the sophistication of the analytic software including accurate compartmentalization of tissue layers, and the availability of conductivity index and electromagnetic properties of each tissue layer for each subject (Wagner et al., 2007). This approach also does not account for real-time state-dependant dynamical changes in tissue excitability captured by EMG and EEG recording. Finally, for the EEG-guided approach to be utilized in real-time, TMS-EEG data should be processed and artifact corrected online. As it stands and discussed later, the offline processing of TMS-EEG data still remains a challenge. Therefore, users should employ each of these methods with care and considering advantages and limitations of each method.

##### Location

Methods of TMS coil placement were discussed in Section Stimulation Location. Similar to adjustment of TMS intensity, when using hot spot of peripheral muscles to identify stimulation location, TMS should be administered over the EEG cap. In non-motor regions, adjustment of coil orientation may not be as obvious in absence of EMG outcomes to guide the coil orientation. In such cases, coil placement can be standardized by fixing the coil angle relative to the gyrification of the underlying cortex in each subject. The utility of neuro-navigation, availability of individual's MRI/fMRI, and ultimately real-time EEG outcomes may enhance the precision of coil orientation and its reliability across stimuli and protocols.

##### Number of stimuli

While TMS-EMG protocols roughly involve 5–20 stimuli per condition (Rossini et al., 2015), TMS-EEG studies often opt out for a much higher number of stimuli. This is often in the range of ~50–200 stimuli per condition to account for the lower signal to noise ratio of EEG (μV amplitude) relative to EMG (mV) outcomes. In general, the accuracy of EEG and EMG outcomes increase with the number of stimuli. However, the optimal number of stimuli required to achieve reliable outcomes may vary as a function of sensitivity of each specific EEG outcome measures, and the quality and reliability of TMS-EEG recording (e.g., impedance, coil placement) for a given session, subject, and brain region.

##### Brain state

The TMS protocols that were introduced using EMG outcome measures are often presented at rest or during controlled activation of a peripheral target muscle guided by a force meter (e.g., CSP protocols). In extending TMS protocols to non-motor regions, more investigations are required to quantify the impact of brain state on TMS protocols. Such investigations may then guide the utility of online EEG neurofeedback systems to maintain the desired brain state during administration of TMS protocols.

### EEG analysis

In designing TMS-EEG experiments, the EEG outcome measure (EEG feature) can be selected based on method of analysis, location, and the time at which they are captured (Figure 1 Output). EEG analysis can involve quantification of EEG signals in terms of amplitude, frequency, phase, the interaction between these attributes, the direction of information flow, and the dynamics of EEG topography, chronometry or tomography (Amico et al., 2015). EEG features may be extracted from one or more sensors or sources. Finally, the EEG features can be described relative to the time of TMS application or change in the brain state.

EEG analysis is often based on the assumption that the EEG signal represents a linear dynamical system. A dynamical system is described by its *state*—the values of all the variables that describe the system at a given time *t*—and its *dynamics*—the laws that describe how the *state* of the system changes over time. By presenting the state of a system by all its *k* variables in a k-dimensional space, the state space is obtained for each given point in time. To obtain the evolution of the dynamical system over time (*t*), the state-space of time points are connected, creating a trajectory of the system. The dynamical system is considered linear if the equations that describe the system are all linear. When considered a linear dynamical system, the EEG signal can be decomposed into Fourier series (Dietsch, 1932), i.e., sine waves described by *amplitude, frequency*, and *phase*. In this model, *amplitude* represents the maximum vertical peak of the sine wave (unit of μV), *frequency* is the number of complete cycles per second (unit of Hz), and *phase* describes the time point position with respect to the beginning of the sine wave [unit of radian or degrees, ranging from −180° (−Π) to 180° (Π)]. To obtain the frequency and phase component, the EEG time series is multiplied by a transfer function, such as Fast Fourier Transform (FFT) or discrete wavelet transforms. In this procedure, a complex number is identified that can be used to compute the instantaneous power (proportional to the square of the maximum amplitude that the signal could reach) and phase of the signal.

The EEG signal can also be considered as a non-linear, stochastic or deterministic, and dissipative dynamical system (reviewed in Stam, 2005). In a non-linear dynamical system, described by non-linear equations, a small change in initial conditions may cause a large effect. In non-linear EEG analysis, chaos theory may be applied to reconstruct an attractor (convergence of trajectories to a subspace) from the EEG time-series. The attractor is described by its dimension, Lyapunov exponents, and entropy (Stam, 2005). The non-linear EEG analyses were employed to describe non-linear synchronization between brain regions and network nodes.

EEG is analyzed at different spatial scales, from single sensor (source) analysis to integration of all sensors (sources). Several mathematical frameworks are developed to reconstruct the sources that underlie the scalp recordings (Pascual-Marqui et al., 1994), a process referred to as solving the *inverse-problem*. The sensor analysis produces a two-dimensional representation of brain activity, while source analysis reveals a three-dimensional representation of the brain activity. There are an infinite number of possible solutions to the inverse problem for scalp EEG topography, and numerous parametric and non-parametric techniques are proposed to solve the inverse problem (Grech et al., 2008).

EEG analyses can be grouped into general categories of reactivity and connectivity analysis. The aim of reactivity analysis is to characterize the regional or global brain response to an event or change in brain state. In these analyses, EEG signals are often characterized by (1) *temporal analysis*: Identifying time domain features including latency and amplitude of *event-related potentials (ERP)*s or *evoked potentials (EP)*s and *Global Mean Field Amplitude (GMFA;* Lehmann and Skrandies, 1980), (2) *frequency analysis:* Decomposing the time domain signals into frequency sub-bands including delta (~1–3 Hz), theta (~4–7 Hz), alpha (~8–12 Hz), beta (~13–28 Hz), and gamma (~>30 Hz) oscillations, and identifying outcome measures such as *evoked* and *induced power, relative* and *absolute power*, or *event-related synchronization (ERS)* or *desynchronization (ERD;* Pfurtscheller and Lopes da Silva, 1999), (3) *time-frequency analysis:* Performing spectral decomposition using a *sliding* time window to calculate the change in power of each frequency as a function of time, thereby, revealing time and frequency domain information and identifying outcome measures including *event-related spectral perturbation (ERSP;* Makeig, 1993), and (4) *phase analysis:* Identifying the phase of the EEG signal at a specific time point or relative to an event.

The aim of connectivity analysis is to describe how two or more functional units, such as two or more brain regions, network nodes/hubs, or brain dynamics (e.g., oscillatory activity) interact, such as function in “synchrony,” to form a larger-scale functional unit that underlies a specific brain-state (Brown and Kocarev, 2000). Connectivity techniques fall within two broad classes. The most commonly used are measures of undirected connectivity (i.e., without quantification of the direction of information flow), including *correlation, coherence*, or *synchrony*. These describe the relationship between signals recorded across the sensors (or sources), and/or across trials, by quantifying the interaction between signal attributes such as amplitude, frequency, and phase. Numerous connectivity and network dynamic metrics can be realized by quantifying the interaction between EEG features across brain regions. The second set of measures can capture the direction of information flow, but can be computationally complex, and are applied to EEG data recently. These are based on the Granger causality principle (Granger, 1969) such as the *directed transfer function* and *partial directed coherence*. As a cautionary remark, the validity and reliability of EEG markers of functional connectivity should be examined against simulated data. Studies suggest that some connectivity analyses are confounded by the effects of volume conduction and are sensitive to the methods of temporal filtering and source reconstruction (Haufe et al., 2013).

To date, TMS-EEG studies have employed ERP (TEP), frequency-domain power (e.g., induced/evoked/relative/absolute power, ERS, ERD), time-frequency (e.g., ERSP), and phase-domain analyses. The latency of TEPs was replicated across several studies (reviewed in Komssi and Kahkonen, 2006). When single-pulse TMS is applied to motor cortex, it generates a negativity at 15 ms (N15), a positivity at 30 ms (P30), followed by N45, P55, N100, P180, and N280 (reviewed in Komssi and Kahkonen, 2006). Some studies investigated the slope of the EEG peaks (Vyazovskiy et al., 2009; Huber et al., 2012). Moreover, the global brain response to TMS can be characterized through GMFA (reviewed in Komssi and Kahkonen, 2006). The frequency-domain analysis of TEPs was employed to examine the modulation of the amplitude or power of specific frequency bands within a fixed-length time-window following the TMS pulse (e.g., Paus et al., 2001; Farzan et al., 2009). Alternatively, *sliding time-windows* were used to characterize TMS-induced changes in the time-frequency domain (e.g., Rosanova et al., 2009; Frantseva et al., 2012; Vernet et al., 2012). Similarly, phase-domain analyses are employed (e.g., Dugue et al., 2011; Stamoulis et al., 2011). Essentially, any methods of EEG feature extraction can be applied to TMS-EEG data.

## Factors affecting TMS-EEG outcome measures

Several factors and parameters can impact TMS-EEG outcomes (Chipchase et al., 2012). These factors should be controlled or accounted for. Broadly, these can be divided into *stimulation parameters, tissue properties, brain state*, and EEG *artifacts*.

### Stimulation parameters

The pulse intensity (Hess et al., 1987; Di Lazzaro et al., 2003), coil orientation (Wagner T. et al., 2004; Pell et al., 2010), coil shape (Di Lazzaro et al., 2004), coil material and manufacturer (Thielscher and Kammer, 2004), and the TMS pulse shape (Di Lazzaro et al., 2001; Sommer et al., 2006) influence TMS outcome measures (see Fox et al., 2004; Pell et al., 2010; Thielscher et al., 2011). These factors should be carefully selected, kept consistent across subjects or multi-site studies, and reported in detail.

### Tissue properties

TMS outcome measures are influenced by the coil-cortex distance and head morphology (Rudiak and Marg, 1994), tissue morphology, white matter anisotropy (Opitz et al., 2011), and the thickness and distribution of the CSF compartment (Wagner T. A. et al., 2004; Bijsterbosch et al., 2012). The site of maximal TMS induced brain activation is not necessarily the site with the shortest distance to the coil (Bijsterbosch et al., 2012). The maximal current may be accumulated on brain tissue covered by the largest amount of CSF fluid, surrounded by CSF thin gyral peaks (Bijsterbosch et al., 2012). This is critical as the CSF volume and distributions are not necessarily the same between the hemispheres and across brain regions (Bijsterbosch et al., 2012), or in patients with traumatic brain injury (TBI; Wagner et al., 2006, 2008). While some of these factors (e.g., coil-cortex) can be more easily controlled for (e.g., by adjusting voltage), others (e.g., CSF volume) cannot be easily and readily controlled for with current technologies. However, these factors should be carefully considered when designing studies in patients (e.g., TBI) and comparing outcomes between patients and controls.

### Brain state

Monitoring and controlling the brain state to enhance the specificity of the TMS impact is among the added values of TMS-EEG methodology. The brain state can be described by at least four components: developmental state (e.g., age), behavioral state (e.g., unconsciousness, sleep, wakeful resting, cognitive processing), circadian and sub-second rhythms (e.g., millisecond changes in spatiotemporal dynamics), and health state (e.g., healthy or a disease state). The TMS outcomes are affected by the brain state at the time of stimulation. There are clear differences in TMS effects across brain developmental state (Garvey and Mall, 2008; Pascual-Leone et al., 2011), behavioral state (Bertini et al., 2004), and transient dynamical state (Silvanto et al., 2008; Morishima et al., 2009). Furthermore, several interventions such as pharmacological interventions (Ziemann, 2004; Premoli et al., 2014), physical exercise (Fowler et al., 2010), cognitive training (Radhu et al., 2012), and neurostimulation (e.g., Farzan et al., 2016) can modify the brain state and hence likely the TMS outcome. In envisioned future TMS equipment, TMS input parameters could be adjusted dynamically as a function of brain state. For example, the sub-second brain dynamics could be quantified through online EEG analysis, and TMS-EEG input parameters could be adapted online to drive stimulation to achieve the desired physiological impact (Rotenberg, 2009; closed-loop system; Figure 2 Brain State).

### Artifacts

Several factors may contaminate acquisition of clean EEG recording. In TMS-EEG studies, EEG outcome measures are affected by artifacts and confound common to EEG recordings in general, and importantly by TMS-related artifacts.

#### Common EEG artifacts

Two general categories of common EEG artifacts are the environmental and physiological noise. The power line noise (50 or 60 Hz) and slow drifts in the electrode position represent environmental noise, often removed offline through digital filters. However, filtering procedures also eliminate physiological information contained in the filtered frequency bands. Furthermore, filtering may impact EEG features that are based on phase-sensitive estimators (e.g., phase-delay). To remedy this, zero-phase shift filters may be used offline. Physiological noise includes physiologically induced signal such as eye blink and movements, cardiac rhythms, head movement, and muscle contraction. To eliminate physiological artifacts, electrooculogram (EOG), and electrocardiogram (EKG) signals could be concurrently recorded with EEG, and assessed offline to guide manual or semi-automated noise removal. More recently, independent component analysis (ICA) is used to project out these sources of noise that typically have distinct temporal, spectral, or spatial characteristics compared to EEG sources.

#### TMS-related artifacts

Despite advances in TMS-EEG equipment, and specifically in EEG amplifier technology, the recovery of the early TMS-induced brain response (~first 50 ms) remains a challenge due to residual short-lived high voltage TMS-induced electromagnetic pulse artifact (Ives et al., 2006) and several other TMS-related *physiological* and *instrumental* noise (see Rogasch et al., 2014; Vernet and Thut, 2014; Ilmoniemi et al., 2015). These factors also include (1) TMS-induced activation of the peripheral nerves and cranial muscles near the coil (TMS-induced EMG artifact; Korhonen et al., 2011; Maki and Ilmoniemi, 2011; Mutanen et al., 2012), (2) movement of the EEG sensors due to the electromotive forces (Sekiguchi et al., 2011), coil vibration with each pulse (Ilmoniemi and Kicic, 2009), or TMS-induced nerve and muscle activation, (3) TMS-induced accumulation of charges and their slow decay at every interface with capacitive properties, including the skin-electrodes interface (Veniero et al., 2009) or even the interface between several deeper epithelial layers of the skin (Julkunen et al., 2008; TMS-induced decay artifact), and (4) the capacitor recharge in biphasic TMS stimulators. Furthermore, (5) the loud clicking sound of each TMS pulse (~100 dB, 0.5 ms rise time; Starck et al., 1996), and (6) the TMS-induced tapping sensation on the scalp generate AEP and SEP (auditory and somatosensory-evoked potentials; Nikouline et al., 1999, 2000), respectively. Finally, TMS can evoke (7) blinks (time-locked to the stimulation) and (8) attentional/arousal effects.

### Spatial resolution of TMS-EEG methodology

Before reviewing how artifacts and variability can be minimized in TMS-EEG studies, we briefly discuss a general short-coming of TMS-EEG methodology: EEG spatial resolution. EEG mainly captures neural activity of the cortical neurons. Thus, direct recording from subcortical structures is not available with scalp EEG. Concurrent combination of TMS-EEG with other neuroimaging modalities such as fMRI (Bohning et al., 1999; or others reviewed in Wagner et al., 2007) or multi-channel cortical and subcortical electrophysiological recording through implanted electrodes may be ways to improve TMS-EEG spatial resolution. However, this latter is only available in a selected few patients with specific disorders or illnesses that require implants. Even in such cases, TMS is often considered unsafe due to potential interaction between the implant and the electric field and risk associated with movement of the implants. While the concurrent combination of TMS with fMRI was made possible in recent years (Bohning et al., 1999), the technique suffers from low temporal resolution. This is a key short-coming given the millisecond resolution of TMS protocols and how they activate different neural circuitries with millisecond accuracy (e.g., as in paired-pulse protocols). Even if TMS-EEG is combined with concurrent fMRI (fMRI and TMS-compatible systems are commercially available), the poor temporal resolution of fMRI would still not permit localizing with accuracy the short-lived TEPs. One should also consider the feasibility of this multimodal setup and its added value by evaluating the participants' comfort, induction of various sources of noise in EEG due to fMRI and TMS, and cost associated with fMRI scanning. We highlight that compared to EEG alone, TMS-EEG has higher spatial resolution given the precision of TMS in activating cortical regions and even specific neurons. The concurrent combination of single neural recording and TMS in awake primates has recently illustrated the specificity of TMS in activating selective neurons (Mueller et al., 2014). Therefore, collectively, while spatial resolution remains a major short-coming, several advantages of TMS-EEG methodology such as temporal resolution, cost, and compatibility with fMRI make it a viable solution for a wide range of applications in health and disease.

## Preventing TMS-related EEG artifacts

In TMS-EEG studies, special attention should be paid to avoid or attenuate and remove TMS-related artifacts. TMS-related artifacts may be avoided or minimized online. Offline, noise removal techniques are used to extract residual artifacts. The following considerations can minimize noise induction during data acquisition:

### Sensor placement

The electrodes that are directly underneath the TMS coil are more prone to TMS-induced artifacts such as eddy currents or sensor movements (including coil movements by the operator). Therefore, the placement of reference and ground sensors near the TMS coil should be avoided.

### Sensor wire arrangement and orientation

In high density EEG recording, it is recommended that (a) sensor wires are kept free of loops to avoid induction of eddy currents; (b) the loose part of the sensor wires are grouped together (e.g., braiding every four electrode wires) toward the amplifier and oriented away from the TMS coil cable to avoid additional interference (Veniero et al., 2009); (c) sensor wires are oriented perpendicular to the TMS-induced current orientation (Sekiguchi et al., 2011) such as perpendicular to the handle of a figure-of-eight coil.

### Sensor-skin and skin-skin impedance

A sensor-skin impedance of < 5 kΩ is desirable. The impedance in the outer epithelial layers should be lowered by cleaning the electrode-skin contact surface, e.g., by scrubbing the skin with alcohol using a cotton-tip swab before applying the electrode paste. The capacitive properties of the deeper epithelial layers can lead to charging of the skin after the pulse, resulting in a slow after-discharge that contributes to the long-lasting TMS artifact (Julkunen et al., 2008). In order to further reduce the skin capacitance and resistance, Julkunen et al. proposed to short-circuit the epithelial layers via a mini-puncturing technique applied to the skin at the sensor contact through the sensor hole. The mini-puncture preparation led to a similar spatial spreading of the artifact, but artifact amplitude and recovery-time were significantly reduced (Julkunen et al., 2008).

### Controlling for TMS click

Each TMS pulse is accompanied by a loud clicking sound, approximately 100–120 dB with a rise of time of < 0.5 ms (Starck et al., 1996). The TMS click produces an AEP time-locked to the TMS pulse delivery (Nikouline et al., 1999; Rogasch et al., 2014). The AEP is induced by the TMS click sound conducted through air but also through the vibration of the temporal bone, bypassing the middle ear and activating the cochlea directly (Nikouline et al., 1999). This TMS-induced AEP is associated with an EEG component that has a negativity at 100 ms and a positivity at 180 ms (N100-P180 complex) prominently observed in the central and parieto-temporal sensors (Nikouline et al., 1999; Rogasch et al., 2014). The amplitude of this component is highest when the TMS coil is held in direct contact with the scalp, and is attenuated when the coil is held 2 cm above the scalp resting on a plastic piece, and further decreased when the coil is held 2 cm above the scalp without direct contact with the scalp (Nikouline et al., 1999).

To attenuate the air-conducted AEP during data acquisition two general approaches are suggested: using hearing protections (e.g., earplugs) or playing loud (~90 dB) white noise through inserted earphones (Paus et al., 2001; Fuggetta et al., 2005). Some investigators use adapted noise by matching the pressure level of each frequency of the applied noise with the frequency of the time-varying magnetic field (Ferrarelli et al., 2010). To attenuate the bone-conducted AEP, a thin layer of foam can be inserted between the TMS coil and the scalp (Massimini et al., 2005). The efficacy of the above-mentioned masking strategies on the N100-P180 component was compared (Ter Braack et al., 2013). It was found that using the combination of a layer of foam and adapted noise was most effective in reducing the amplitude of N100-P180 component. However, it was shown that the N100-P180 component was present in deaf subjects, suggesting that this component is a mix of direct brain activation and AEP. Alternatively, AEP can be removed offline. For example, TMS-induced potentials in a control condition (e.g., sham TMS) can be subtracted from the potentials in the corresponding active condition in offline data analysis (Daskalakis et al., 2008). Another approach involves the utility of component analysis (e.g., ICA Rogasch et al., 2014) to extract and remove AEP components.

### Controlling for scalp sensation

A TMS pulse may result in activation of scalp tactile receptors, scalp muscles, or the trigeminal nerve terminals directly, producing SEP. The topography of the TMS-induced scalp sensation is suggested to be asymmetrical, peaking ipsilateral to the stimulation site (Nikouline et al., 1999). To attenuate SEP, different TMS coil orientations may be used as control conditions. However, it is important to remember that the effects of TMS vary with the TMS coil orientation (Bonato et al., 2006; Thut et al., 2011a). The coil-sensor wire angle should be kept constant to not alter the TMS-induced electromotive forces (Sekiguchi et al., 2011).

### Controlling for attention

EEG is sensitive to changes in attention. To control for cross-experiment and cross-subject differences in attention, participants' attention may be systematically oriented toward a task. In a study by Huber et al. (2008), subjects were engaged in a simple oddball task. However, in many experimental designs, such distracting tasks might be a problem, as engaging in a task likely alters the brain state and brain network connectivity, which in turn may influence the TMS-EEG outcomes (Morishima et al., 2009; Miniussi and Thut, 2010).

### Other control conditions

In experiments where TMS-EEG is applied during task performance, the control condition can be the identical TMS protocol without the task. The EEG potentials in the no-task condition can then be subtracted from the task condition (Thut et al., 2003, 2005). However, the TMS-evoked response common to both conditions will be also eliminated. Other investigators have simply used sham TMS (Daskalakis et al., 2008; Voineskos et al., 2010; e.g., sham coils or tilting the active coil 90 degrees). However, the available sham stimulation paradigms do not closely mimic the sensations associated with real TMS (Davis et al., 2013). Stimulating other brain areas or delivering the stimulation at other timings, with other frequencies, or intensities can also be used to control for non-specific TMS effects.

## Correcting TMS-EEG artifacts

Despite the meticulous application of online noise reduction techniques, preventing EEG contamination by common and TMS-related artifacts is not always fruitful. Therefore, EEG data analysis often requires a dedicated preprocessing step for removing various artifacts. TMS-EEG signal processing follows three general steps of data preprocessing, data analysis and statistical analysis. The main difference with no-TMS EEG analysis is removal of residual TMS-related artifacts. In particular, TMS-related artifacts can contaminate the first 50 ms of TEPs, and the slow decay of the accumulated charges may increase this artifact duration. Moreover, TMS-induced and time-locked physiological artifact such as TMS-induced EMG, eye blinks or movements, AEP, SEP, and trigeminal nerve stimulation, can mask the TEPs. The offline removal of the TMS-induced artifact and the preservation of the early TMS-induced brain responses still pose unresolved challenges. The two general approaches in removing artifacts from TMS-EEG data include: deletion of contaminated section (signal + noise) from analysis vs. attempts to preserve early EEG potentials by employing mathematical techniques to disentangle the brain response from a mixture of TMS-induced artifacts.

### Removing data contaminated with artifacts

The purpose of this approach is to minimize the presence of the large-amplitude early TMS pulse artifact or TMS-related EMG artifacts that can cause data smearing in other analysis steps.

#### Removing sensors

The simplest approach is excluding data from contaminated sensors (Kahkonen et al., 2001). If large numbers of sensors are affected, this approach may not be feasible. Since the sensors closest to the stimulation site are contaminated with higher probability, this approach may create a regional bias. This may violate the underlying assumptions (e.g., symmetrical brain coverage by EEG sensors) of other steps such as average re-referencing or source reconstruction (Litvak et al., 2007). To avoid this asymmetry, the method of spherical interpolation may be used to replace the excluded sensors (Litvak et al., 2007; Hamidi et al., 2010).

#### Removing trials

Another strategy is to exclude contaminated trials in a sensor-specific manner to preserve the most number of sensors (Reichenbach et al., 2011). However, this approach is prone to bias as more trials are likely deleted in the vicinity of stimulation site, resulting in lower signal-to-noise in nearby sensors. To minimize bias, a threshold may be set to avoid deletion of more than a specific fraction of trials per sensor. Furthermore, for each trial, noisy sensors may be removed and interpolated to avoid deleting the entire trial. In this scenario, likewise, a pre-set threshold may be used to delete the entire trial once the number of noisy sensors in that trial passes a specific threshold (e.g., if >20% of sensors were noisy in the trial).

#### Removing the contaminated time segment

Another alternative or complementary approach involves cutting or replacing the contaminated time period. This can be conducted in a sensor-, trial-, and subject-specific manner to preserve the maximal amount of data, or using a time-window of fixed-length equivalent to the average duration of the TMS artifact across trials or subjects (Daskalakis et al., 2008). For example, in one study time window −2 to 38 ms relative to the TMS onset was cut, the pre- and post-stimulus data were concatenated, and the random voltage step between the joined data points of each single trial were eliminated by averaging the trials (Fuggetta et al., 2006). Data concatenation should be carried out with care as edge artifacts were observed due to data discontinuity (Fuggetta et al., 2008). Other approaches are to set the value of the removed section to zero (Esser et al., 2006; Van Der Werf and Paus, 2006; Huber et al., 2008), or to interpolate the missing data (Reichenbach et al., 2011; Garcia Dominguez et al., 2014).

### Recovering the brain signals

The purpose of this approach is to selectively remove the TMS-related artifacts from the brain response.

#### Template subtraction

A few studies have examined the effect of various TMS stimulation parameters on the amplitude and duration of TMS-induced artifacts when TMS is applied to a human compared to a dummy head (Bender et al., 2005; Veniero et al., 2009). These studies attempted to capture the profile of TMS artifact using phantom head models with the same EEG electrodes, cables, and TMS coil so that the artifact can be subtracted from the real recording (Bender et al., 2005; Veniero et al., 2009). However, in practice, these setups cannot fully separate brain signals from noise as they are not identical to the TMS-EEG experiments (e.g., phantoms used are not identical to human heads). Assuming that the shape and duration of the TMS-induced early artifact remain the same for each EEG sensor in a given subject, a “calibration trial” can be performed to obtain an artifact template for each EEG sensor. This template can then be used to remove the artifact from the real condition by subtraction (Thut et al., 2003, 2005; Fuggetta et al., 2006; Reichenbach et al., 2011). This method is frequently applied in studies involving a task, and the calibration trial involves applying the same TMS-EEG condition without the task. In this setting, synchronization boxes are particularly important to improve the reproducibility of the artifacts and thus the validity of a template artifact.

#### Recursive prediction-correction filters (bayesian model)

A shortcoming of the template subtraction is the general assumption that the TMS artifact is stationary. However, TMS-related artifacts may not be reliably reproduced following each stimulus due to noise and signal non-stationarity. To account for this, the suitability of a Bayesian model for removal of the TMS-induced artifact was investigated by linear Kalman filters (Morbidi et al., 2007). A Kalman filter is a prediction-correction algorithm that operates recursively on a series of noisy input data to produce statistically optimal estimates of the underlying system state by computing the linear minimum mean squared error. A disadvantage of Kalman filter is that it has to be based on a state model that can describe the mechanism underlying the signal generation (Morbidi et al., 2007). Such models that could fully account for the many factors contributing to the brain states dynamics are not yet available.

#### Blind source separation

The purpose of this approach is to separate out noise by using mathematical procedures and decomposing EEG signals into independent components (e.g., via independent component analysis, ICA) or orthogonal components (e.g., via principal component analysis, PCA). Schematically, components associated with noise are removed and then noise-free physiological components are mixed to reconstruct the data back to the sensor level. Following an ICA approach, components reflecting noise are often identified based on their spatial and temporal features. The validity and outcome of ICA in removing TMS-related artifacts were documented for DLPFC and motor cortex stimulation (Rogasch et al., 2014). PCA can be applied to a specific time window or frequency band that is predominately contaminated by noise (e.g., EMG; Maki and Ilmoniemi, 2011). PCA components can be also used to feed models estimating artifact and brain signal topographies (Litvak et al., 2007; Levit-Binnun et al., 2010).

Unfortunately, common to all these artifact correction techniques, including the blind source separation, is the questionable assumption that the brain signals and TMS artifact are spatially or temporally independent or can be orthogonalized. As examples, while ICA has proven successful in removing EOG artifact, TMS can induce time-locked eye blinks or AEP that overlap with TEP components of interest such as N100 component. A recent study examined the performance of three different ICA algorithms for separating out the time-locked EOG artifact. The ICA algorithms include: Fast ICA, which maximizes non-Gaussianity of the source components, Run ICA, which minimizes the mutual information between the sources, and the temporal decorrelation source separation (TDSEP), which utilizes temporal information in the source signal. It was shown that TDSEP performed better than Fast ICA and Run ICA in removing the ocular artifacts from TMS-EEG signals (Lyzhko et al., 2015). Moreover, the large-amplitude TMS-related artifacts may further lead to poor ICA performance. Various methods are proposed to circumvent these challenges, such as suppressing the amplitude of TMS-related artifact (Hernandez-Pavon et al., 2012), applying a pre-processing step to uncover hidden components by mean-subtraction (Metsomaa et al., 2014), or removing the TMS-related large amplitude decay artifact through application of a preprocessing ICA step prior to the main ICA run, to first project out the large-amplitude artifact component (Rogasch et al., 2014).

**Figure 3:**
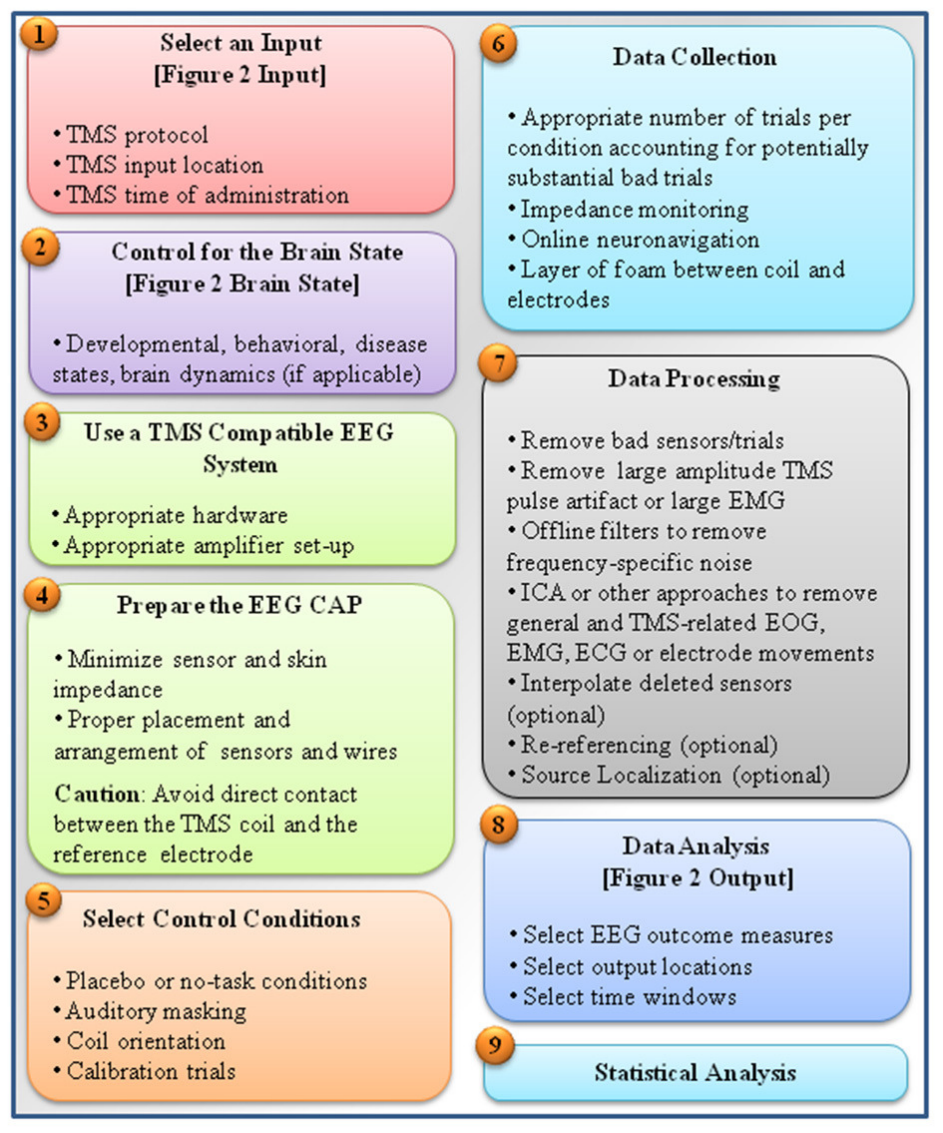
**A summarized step-by-step guideline for carrying out a TMS-EEG experiment**. The figure is a summary of major steps taken in conducting a TMS-EEG experiment. These steps include: (1) selection of TMS input parameters (e.g., input location, protocols, and time) as depicted in Figure 2 and described in Section TMS Parameters, (2) making note of or controlling the brain state as described in Section Brain State, (3) choosing TMS-compatible EEG systems as described in Section TMS-EEG Equipment, (4) proper EEG cap preparation for minimizing induction of TMS artifacts as described in Section Sensor Placement, Sensor Wire Arrangement and Orientation, and Sensor-Skin and Skin-Skin Impedance, (5) incorporating appropriate experimental conditions as control conditions described in Section Controlling for TMS Click, Controlling for Scalp Sensation, Controlling for Attention, and Other Control Conditions (e.g., including sham conditions, masking TMS loud clicking sound, and vibration), (6) following several considerations during data acquisition to prevent induction of noise (e.g., placement of a layer of foam between coil and electrodes) or to properly adapt TMS protocols and enhance signal to noise ratio by using large number of stimuli per condition as described in Section Implementation of TMS Protocols during EEG Recording and Preventing TMS-Related EEG Artifacts, (7) following recommended guidelines for data processing, involving first removing large amplitude TMS-related artifacts such as TMS pulse artifact or TMS induced EMG before application of filters as described in Section Correcting TMS-EEG Artifacts, (8) selection of appropriate EEG output parameters (e.g., input location, protocols, and time) as depicted in Figure 2 and described in section EEG Analysis, and finally (9) choosing an appropriate statistical framework suitable for the characteristics of the multidimensional TMS-EEG outcomes (e.g., based on the data dimensions, or distribution characteristics; Frehlich et al., 2016) also described in Section Correcting TMS-EEG Artifacts.

Integrating what was discussed thus far, Figure 3 provides a step-by-step guideline for carrying a TMS-EEG experiment, from selecting TMS-EEG parameters to carrying out TMS-EEG artifact correction and analyses. Several important points must be emphasized. First is employing all measures possible to prevent induction of TMS-related artifacts in the first place. The second point is removing large-amplitude TMS-related artifacts early on in the preprocessing step, and definitely prior to application of filters. It is highly recommended that researchers report all the discussed parameters involved in TMS-EEG data processing (including the order of artifact correction). This is a step toward ensuring that across laboratories and studies TMS-EEG outcomes can be replicated and integrated. Finally, as it is common to analysis of any multidimensional brain data, appropriate statistical frameworks must be used to account for the problem of multiple comparisons (e.g., see Maris and Oostenveld, 2007; Frehlich et al., 2016).

## TMS-EEG applications

**Figure 4:**
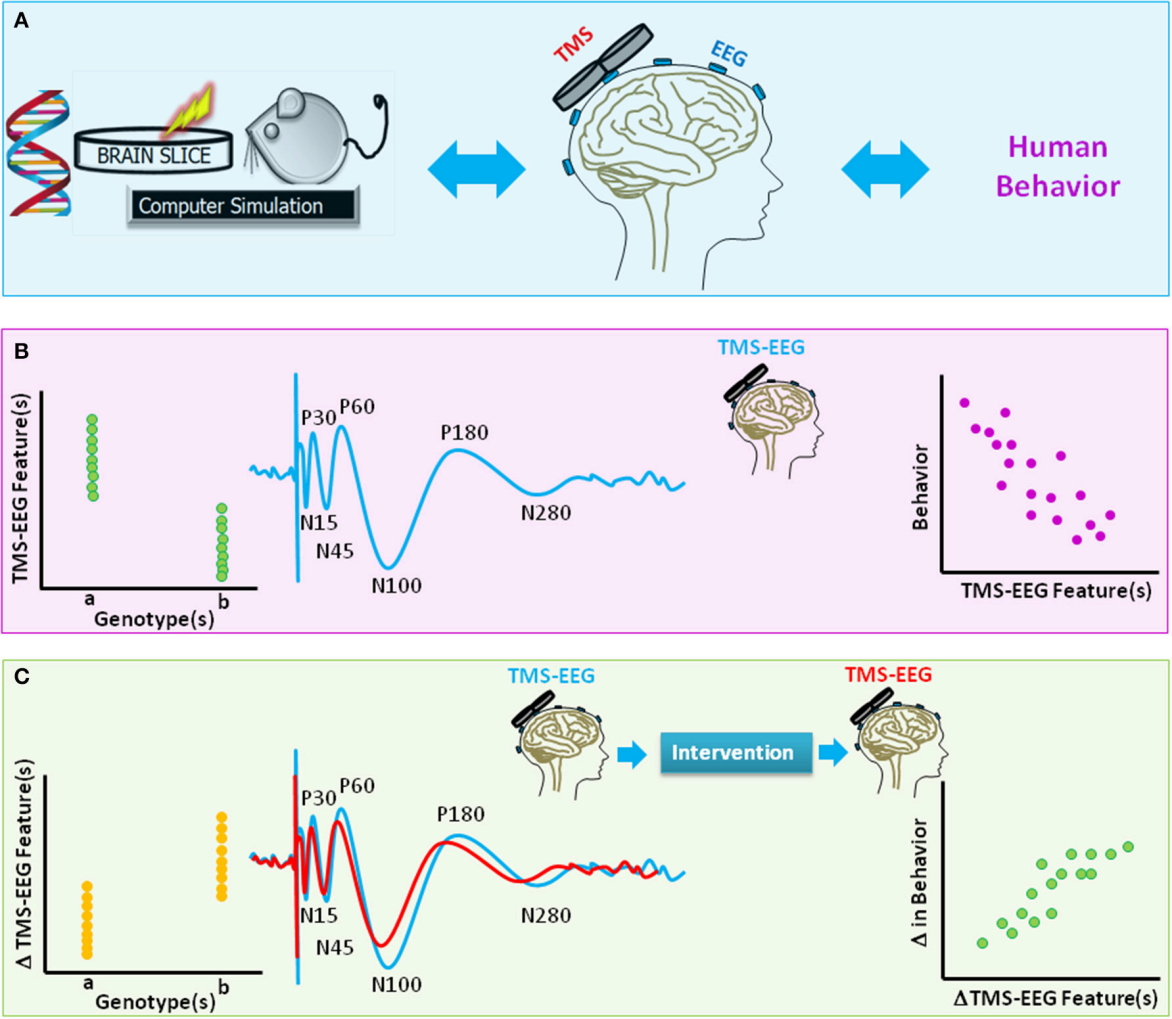
**A schematic diagram of translational value of TMS-EEG. (A)** The method of TMS-EEG provides a means to *non-invasively* assess the integrity and characteristics of numerous brain circuitries in the *intact* human brain using an input (TMS)-output (EEG) approach. Equivalent *in vitro*, animal, genetic, or computational modeling studies can further provide insight into the link between genes, brain function, and behavior. **(B)** The blue waveform illustrates schematics of typical TMS-evoked potential (TEP) when suprathreshold single-pulse TMS is applied to the primary motor cortex. Various characteristics of TEP such as amplitude or latency of components [e.g., negativity at latency 15 ms (N15), positivity at latency 30 ms (P30), N45, P60, N100, P180, N280] are highlighted. The scatter plots are schematic illustrations of the link between TMS-EEG features and genetic variations (left panel) or behavior (right). **(C)** The waveforms highlight change in TEPs for two hypothetical brain states (e.g., before and after an intervention). The scatter plots are schematic illustrations of the link between change in TMS-EEG features and genetic variations (left panel) or change in behavior (right panel).

TMS-EEG methodology has applications in basic science, cognitive neuroscience, and clinical research (Figure 4). TMS-EEG permits *in vivo* assessment of neural excitation, inhibition, connectivity, and plasticity across brain regions and brain states and, thereby, provides fundamental insights into brain function and dynamics, as well as the brain-behavior relationship in health and disease. Importantly, TMS-EEG can be utilized to identify neurophysiological impairments common across brain disorders (Canali et al., 2015), an initiative promoted by the National Institute of Mental Health in the USA. Genetic studies in humans have begun to evaluate the genetic basis of inter-individual differences in TMS-EEG markers of brain health (Lett et al., 2016). Thus far, clinical research studies indicate that TMS-EEG markers have tremendous predictive, diagnostic, and prognostic potential across neuropsychiatric disorders (e.g., Casali et al., 2013; Ragazzoni et al., 2013; Kimiskidis, 2016; Sun et al., 2016).

In basic science and translational research, TMS protocols are adapted to rodents (Vahabzadeh-Hagh et al., 2012) to probe brain disorder mechanisms. For example, a paired-pulse protocol equivalent to LICI was adapted for anesthetized and awake rats (Vahabzadeh-Hagh et al., 2011; Hsieh et al., 2012) where the dependence of this phenomenon, at least in part, on the GABA_A_ receptor was demonstrated. Furthermore, dependence of an LTD-like low frequency rTMS effect on the NMDA-type glutamate receptor was identified in rats (Muller et al., 2014). Specifically coupled with EEG, TMS has not yet been extensively studied in rodents, though in two protocols TMS-EEG enabled measures of the rTMS anticonvulsant effect in a rat seizure model (Rotenberg et al., 2008). Furthermore, simultaneous TMS and single neuron recordings were achieved in alert non-human primates (Mueller et al., 2014). A particularly appealing feature of TMS-EEG in the context of animal studies is that the methodological approach is identical to the one applicable in humans. Therefore, TMS-EEG can deliver true translational phenotypic biomarkers (see Diester et al., 2015).

*In vitro*, TMS can be utilized to assess the cellular (Vlachos et al., 2012; Tang et al., 2016) and sub-cellular impacts of TMS (Murphy et al., 2016). For instance, sub-cellular recordings using optical fiber imaging revealed that single-pulse TMS caused GABA_B_ mediated inhibition of dendritic activity—a finding with direct relevance to the scalp EEG signal that reflects largely dendritic depolarization (Murphy et al., 2016). *In vitro* studies in isolated brain slices examined the effect of rTMS in hippocampal (Vlachos et al., 2012) or cerebellar slice cultures to confirm that rTMS results in an LTP-like durable increase in glutamatergic synaptic strength (Vlachos et al., 2012), or leads to frequency-dependent modulation of functional connectivity (Tang et al., 2016).

The identification of equivalent markers of neural health across *in-vitro* preparations, animal models, and humans has numerous applications in translational research (Diester et al., 2015), including designs of animal models of brain disorders and assessing the safety and efficacy of novel interventions prior to translation to human clinical trials (Figure 4).

### Basic science

In contrast to standalone EEG or TMS, TMS-EEG allows for investigation of intrinsic and functional properties of brain systems in a more controlled and direct manner. Furthermore, in contrast to classical TMS studies, TMS-EEG permits evaluation of *non-motor* systems across a variety of spatiotemporal and spectral scales. TMS-EEG studies have applied well-established TMS protocols to motor and non-motor regions with the general aim of characterizing EEG measures “equivalent” to the MEP measures. Moreover, several TMS-EEG experiments have gone beyond this and developed novel indices of brain intrinsic and functional properties.

#### Markers of brain health

Collectively, the TMS-EEG studies conducted so far have identified EEG indices associated with inhibitory (e.g., GABAergic) neurotransmission (Nikulin et al., 2003; Bender et al., 2005; Daskalakis et al., 2008; Farzan et al., 2013; Premoli et al., 2014), cortical excitability (e.g., Komssi et al., 2004), cortical plasticity (e.g., Esser et al., 2006; Vernet et al., 2012; Rajji et al., 2013; Veniero et al., 2013), cortical conductivity (e.g., Frantseva et al., 2012), interhemispheric connectivity (e.g., Voineskos et al., 2010), cerebellocortical connectivity (Schutter and van Honk, 2006), effective connectivity (Ferrarelli et al., 2010), and the integrity of the thalamocortical loop (Van Der Werf et al., 2006; Rosanova et al., 2009).

The EEG equivalents of MEP modulations in the single-pulse (Maki and Ilmoniemi, 2010a; Farzan et al., 2013), LICI (Daskalakis et al., 2008), SAI (Noda et al., 2016), 5 Hz rTMS (Esser et al., 2006), 1 Hz rTMS (Casula et al., 2014), continuous theta burst stimulation (cTBS; Vernet et al., 2012), and PAS (Rajji et al., 2013) protocols, were examined. It was shown that TMS-EEG metrics of brain properties, including natural frequency or intrinsic GABAergic activity, vary across brain regions (Kahkonen et al., 2004; Farzan et al., 2009; Rosanova et al., 2009). For example, single-pulse TMS-EEG was employed before and after 1 Hz rTMS applied to motor cortex or V1 area in occipital cortex in healthy subjects (Casula et al., 2014). It was illustrated that 1 Hz rTMS reduced MEP amplitudes, and potentiated TEP components (e.g., P60 and N100) that are suggested to be associated with activation of GABAergic mechanisms. By contrast 1 Hz rTMS over occipital cortex did not result in a change in motor excitability (Casula et al., 2014). The assessment of test-retest reliability of several TMS-EEG indices is underway (Lioumis et al., 2009; Farzan et al., 2010a). The value of TMS-EEG markers of brain reactivity and dynamic is also highlighted by the findings that EEG is more sensitive than EMG in detecting TMS-induced modulation of neural activity. For example, TMS input parameters that do not result in measurable MEP responses resulted in measurable TEPs (Komssi et al., 2004), and that the effects of rTMS on brain oscillations outlasted rTMS effects on MEPs (Noh et al., 2012; Vernet et al., 2012).

TMS-EEG studies have examined the spatiotemporal evolution of TEPs. For example, the spatial propagation of TMS-induced phase-resetting was investigated by applying TMS to the occipital cortex and examining the direction of information flow using a transfer entropy index (Kawasaki et al., 2014). It was shown that depending on the intensity of stimulation, TMS could modify the causal relationships between brain areas.

Furthermore, the neurophysiological correlates of behavioral states (e.g., consciousness, or cognitive performance) were investigated using TMS-EEG. As examples, the breakdown of brain effective connectivity during sleep (Massimini et al., 2005), the influence of anesthetics on the brain spatiotemporal characteristics (Sarasso et al., 2015), and the modulation of excitability as a function of time awake (Huber et al., 2012) were studied using TMS-EEG. Therefore, TMS-EEG provides a means to discover neural markers of brain health and study the brain-behavior relationship.

#### Predicting brain state

A growing line of research is dedicated to investigating the *state-dependency* of brain intrinsic and functional properties. The brain state can be described at different scales (e.g., developmental, behavioral, health, and sub-second and circadian dynamical states). TMS-EEG provides *high temporal resolution* and a means to investigate the impact of *dynamical states* on brain functions. As examples, evidence suggests that the initial state and the recent history of neuronal oscillatory activity may be predictive of the brain response and physiological consequences (e.g., MEPs, phosphenes, TEPs) to TMS (Zarkowski et al., 2006; Sauseng et al., 2009a; Maki and Ilmoniemi, 2010a; Dugue et al., 2011; Bergmann et al., 2012; Kundu et al., 2014).

Several studies have investigated whether the pre-stimulus EEG features could predict the TMS-induced MEP amplitude. Evidence now suggests that local activity and long-range interaction between brain regions, as well as corticomuscular coherence could influence the MEP amplitude. As examples, it was shown that the power of spontaneous alpha oscillations in the sensorimotor cortex immediately prior to administration of TMS was negatively correlated with the amplitude of TMS-induced MEP (Zarkowski et al., 2006; Sauseng et al., 2009a). Furthermore, the phase of the mid-range beta oscillations recorded distally over the occipital cortex correlated with MEP amplitude (Maki and Ilmoniemi, 2010a,b), providing evidence that the oscillatory activity in non-motor regions may influence motor system excitability at rest. Using concurrent TMS-EEG-EMG, the functional connectivity between cortical and muscular activity (corticomuscular coherence) was studied (Keil et al., 2014). It was illustrated that EEG and EMG power and phase at approximately 18 Hz correlated with the MEP amplitude. Furthermore, a linear relationship was identified between the corticomuscular coherence at high beta frequency band (e.g., 34 Hz) and the MEP amplitude. This finding was interpreted as showing that strong synchrony between cortex and muscles may lead to generation of large MEPs (Keil et al., 2014).

Studies are also examining the state-dependency of non-motor areas. For example, it was shown that pre-stimulus alpha power and the phase of alpha oscillations prior to TMS administration to the visual cortex could predict the perception of TMS-induced phosphenes (Romei et al., 2008; Dugue et al., 2011). Therefore, TMS-EEG provides a means to study the causal impact of neural dynamics on the local and distributed brain functions and behavior.

### Cognitive neuroscience

In recent years it has become increasingly apparent that complex brain functions depend critically on dynamical interactions within and between multiple brain networks. By studying these interactions across behavioral states, or in response to an external perturbation, new knowledge may be gained on the generation and modulation of human behavior. TMS-EEG has been used to examine and confirm the *causal relationship* between the brain spatiotemporal dynamics and behavior. One can employ at least two different approaches to examine the brain-behavior relationship with TMS-EEG: (1) offline and (2) online designs.

#### Offline approach

In this approach, the participant's performance in a cognitive task is *assessed, then interfered with, and then reassessed*. In addition to the baseline behavioral assessment, TMS-EEG protocols can be employed in a non-interruptive way during the task to quantify the neurophysiological correlates of the brain state. For example, single-pulse TMS-EEG can be applied to the prefrontal cortex during working memory performance to assess the reactivity of the prefrontal cortex, or spatiotemporal dynamic of signal propagation (Miyauchi et al., 2016). rTMS can then be applied at rest to selectively *interfere* with (induce, suppress, facilitate) the brain reactivity or spatiotemporal dynamics. Immediately after rTMS, the performance on the cognitive task can be *reassessed* and TMS-EEG can be re-employed to identify the link between the neurophysiological and behavioral modifications.

To date, several studies have employed offline (r)TMS and EEG to investigate the brain-behavior relationship (e.g., Evers et al., 2001; Jing et al., 2001; Klimesch et al., 2003; Hansenne et al., 2004; Barr et al., 2009; Rizk et al., 2013; Capotosto et al., 2014; Hanslmayr et al., 2014; Gohil et al., 2016; for a review of (r)TMS effects on task-induced ERP see Thut and Pascual-Leone, 2010; Rego et al., 2012). An example of the offline approach is an rTMS study (Klimesch et al., 2003) which demonstrated that parietal rTMS tuned to the individual's alpha frequency enhanced performance in mental rotation tasks by influencing the dynamics of task-related alpha desynchronization, while rTMS at other frequencies had no effect. In another offline approach (Barr et al., 2009), 20 Hz rTMS applied to DLPFC resulted in selective modulation of gamma oscillations in the DLPFC during subsequent working memory performance. Another study showed that the behavioral consequences of cTBS applied to the right posterior parietal cortex could be predicted by the alpha coherence of the right temporo-parietal cortex before the stimulation (Rizk et al., 2013). Finally, a series of studies have investigated the effect of (r)TMS on cognitive ERP component P300 that is linked to shift of attention, context-updating, and orienting to a deviant stimulus (reviewed in Rego et al., 2012). This growing segment of studies suggest that (r)TMS may have the potential to selectively modulate characteristics of the P300 component and likely the corresponding cognitive processes.

#### Online approach

In this approach, TMS is applied during EEG recording to one or more brain regions (networks) at specific time intervals *during* a cognitive performance to *interfere* with (induce, suppress, facilitate) the features of a functional unit (e.g., neural oscillations, spatiotemporal dynamics) and examine the effect of this interference on the dynamics of the functional unit and behavior. Similar to the offline approach, a baseline assessment may be conducted to *assess* the participant's uninterrupted performance, and to characterize the task-specific functional unit. This baseline assessment can guide the selection of appropriate TMS input parameters. For example, EEG functional connectivity measures (e.g., directed transfer function, partial directed coherence) can estimate the direction of information flow between brain regions during cognitive performance. This information can be used to design a controlled TMS input to selectively suppress, facilitate or induce information flow between regions, thus confirming the causal relationship between network dynamics and behavior.

Several online (r)TMS-EEG studies have applied rTMS or single-pulse TMS during task performance concurrent with EEG recording to elucidate the functional roles of neurophysiological markers (e.g., ERP components) and/or brain regions and networks during tasks such as spatial attention control (Capotosto et al., 2012a,b), inhibition control (Yamanaka et al., 2013), perceptual decision making (Akaishi et al., 2013), goal-directed action (Verhagen et al., 2013), face-processing (Mattavelli et al., 2013), temporal encoding (Wiener et al., 2012), sensorimotor integration (Verhagen et al., 2012), and multisensory processing (Romei et al., 2012).

For example, the online approach was used in healthy humans to examine the role of anterior intraparietal sulcus in integrating an object's spatial (e.g., size) and perceptual features (e.g., softness) during motor planning in a grasping task (Verhagen et al., 2012). It was demonstrated that single-pulse TMS applied to anterior intraparietal sulcus, within 200 ms of object presentation, reduced the electrophysiological correlates of motor planning and impaired subjects' ability to use learned knowledge in movement planning. Wiener et al. (2012) illustrated that a 200 ms burst of 10 Hz rTMS applied to the right supramarginal gyrus prior to presentation of a visual stimulus prolonged the subject's judgment of the visual stimulus duration and modulated the contingent negative variation, an ERP that has been associated with temporal coding. Single-pulse TMS-EEG has been employed during task performance to examine the causal effect of one brain region over others as a function of task type and behavioral performance. For example, single-pulse TMS was applied to the prefrontal cortex during a visual discrimination task to assess the top-down regulation of neural activity in the posterior brain regions by the prefrontal cortex. It was illustrated that the TMS pulse applied to the prefrontal cortex propagated to different posterior visual areas depending on the domain of visual features the subjects attended to (Morishima et al., 2009). Furthermore, the amount of signal transmission was associated with the level of attentional preparation and performance of visual selective-attention tasks (Morishima et al., 2009).

Finally, Thut et al. demonstrated that applying rTMS at a specific frequency could increase the power of brain oscillations within the frequency band, likely via phase alignment of natural neural oscillators to each TMS pulse (Thut et al., 2011a,b). These results suggest potential for rhythmic TMS protocols to entrain functionally relevant brain oscillations and explore online the causal relationship between oscillations and cognition. Several studies demonstrated that applying TMS at alpha, beta or gamma frequencies could modulate spatial attention orientation, conscious visual perception, global vs. local treatment of visual stimuli and short-term memory (Sauseng et al., 2009b; Romei et al., 2010, 2011; Chanes et al., 2013). Recent studies integrated rhythmic stimulation with simultaneous EEG and behavior measurements. Using this approach, one study provided evidence for the causal role of prefrontal cortex beta frequency desynchronization in memory formation (Hanslmayr et al., 2014). It was illustrated that beta (~19 Hz) rTMS, but not ~7 or 11 Hz rTMS or sham, applied to the inferior frontal gyrus impaired memory formation. This study illustrated that only beta rTMS led to lasting beta entrainment post TMS and that the strength of this beta echo was associated with memory impairment (Hanslmayr et al., 2014).

### Clinical applications

TMS-EEG has been used in clinical research to examine the biological deficits underlying brain disorders. It is also used to develop prognostic, diagnostic, and treatment strategies for patients, and individuals at high risk of developing pathologies.

#### Diagnostic

Several TMS-EEG studies have investigated the integrity of brain circuitries in neuropsychiatric conditions across a wide age range. As examples, studies in schizophrenia reported deficits of the TMS-EEG outcome measures associated with generation and GABAergic-mediated modulation of gamma oscillations in the prefrontal cortex (Ferrarelli et al., 2008; Farzan et al., 2010b; Radhu et al., 2014), or slowing of natural frequency of frontal cortical/thalamocortical circuits (Ferrarelli et al., 2012). In geriatric patients with Alzheimer's Disease and cognitive decline, impairments of TMS-EEG indices of cortical excitability and connectivity were observed (Casarotto et al., 2011; Julkunen et al., 2011; Ferreri et al., 2016).

Moreover, TMS-EEG was employed to characterize impairments of inhibitory mechanisms in children with attention deficit hyperactivity disorder (ADHD; Bruckmann et al., 2012) and in adult patients with epilepsy (Valentin et al., 2008; Del Felice et al., 2011; Julkunen et al., 2013; Shafi et al., 2015). The amplitude of the N100 component was smaller and its latency shorter in children with ADHD compared to healthy control children (Bruckmann et al., 2012). In epilepsies, TMS-EEG was used to identify the epileptogenic zone and showed potential for revealing biomarkers of response to invasive neuromodulatory therapies (Rotenberg, 2010; Shafi et al., 2015; Kimiskidis, 2016).

Furthermore, TMS-EEG studies *across* patient populations such as schizophrenia (vs. bipolar disorder and obsessive compulsive disorders; Farzan et al., 2010b; Radhu et al., 2014) or Alzheimer's Disease (vs. mild cognitive impairment; Julkunen et al., 2011) have identified disease-specific impairments in TMS-EEG indices of brain dynamics, providing potential EEG markers to be explored as risk factors, endophenotypes, and biomarkers of a disease or disease-state. Therefore, TMS-EEG has realistic potential in this research domain to be employed as a diagnostic technique to guide stratifications of patients based on common biological deficits.

TMS-EEG measures are also utilized to assess consciousness. As reviewed elsewhere (Sarasso et al., 2014), the complexity of the EEG reactivity to TMS is markedly reduced in subjects (1) during slow-wave sleep (Massimini et al., 2005) but not in REM sleep (Massimini et al., 2010); (2) during infusion of sedative anesthetics such as midazolam (Ferrarelli et al., 2010), propofol, and xenon (Sarasso et al., 2015), but not during infusion of ketamine, an anesthetic which uniquely results in vivid dreams (Sarasso et al., 2015); and (3) in patients in persistent vegetative state, but not patients who are minimally conscious or locked-in (Rosanova et al., 2012; Casali et al., 2013; Ragazzoni et al., 2013). Finally, it was reported that TEPs provided complementary diagnostic value compared to the standard SEP (sensory-evoked potentials) or ERPs in assessment of consciousness (Ragazzoni et al., 2013).

#### Predictors and mechanistic markers of treatments

TMS-EEG measures can be used to predict the therapeutic outcome or evaluate the mechanism of action of a pharmacological agent (e.g., GABAergic), neuromodulation therapy (e.g., rTMS, Electroconvulsive Therapy, transcranial Direct Current Stimulation), cognitive training, physical exercise, or a combination of interventions (Kahkonen et al., 2003; Kahkonen and Wilenius, 2007; Barr et al., 2012; Plow et al., 2012; Casarotto et al., 2013; Romero Lauro et al., 2014; Kimiskidis, 2016). In this context, TMS-EEG is employed as a diagnostic tool to assess brain dynamics and neurophysiology prior to administration of the intervention (prediction markers), and then again following the intervention (mechanistic markers). For example, a recent single- and paired-pulse TMS-EEG study revealed that the TEP N100 component and LICI in the prefrontal cortex prior to a course of magnetic seizure therapy predicted remission of suicide ideation in patients with treatment-resistant depression (Sun et al., 2016). Longitudinal study designs can capture the dose-response curve of the intervention on specific neural mechanisms. In a sham-controlled rTMS study of children with ADHD, suprathreshold single-pulse TMS-EEG was used before and after subthreshold 1 Hz rTMS applied to the primary motor cortex to examine the rTMS-related changes in cortical excitability (Helfrich et al., 2012). It was shown that the amplitude of N100 component was reduced following 1 Hz rTMS. This was suggested to reflect a general reduction in brain reactivity or inhibitory mechanisms (Helfrich et al., 2012). Collectively, these studies highlight the modulatory effect of age and disease state on brain dynamics, and the sensitivity of TMS-EEG in quantifying brain dynamics across the life span and disease states.

#### Guided treatment

Identifying TMS-EEG neurophysiological indices that underlie a disease state could be helpful in designing targeted and individualized therapies. For example, the TMS-EEG findings of frequency- and region-specific gamma oscillations in schizophrenia (Ferrarelli et al., 2008; Farzan et al., 2010b) can guide the development of rTMS protocols applied to the prefrontal cortex (Barr et al., 2012) or other interconnected nodes (Farzan et al., 2012) to relieve impairments. Similarly, the rTMS-modulation of attention-related alpha oscillations in the parietal cortex (Thut et al., 2011a) has potential clinical applications in disorders of attention and neglect. In depression, EEG can guide synchronized TMS therapy tuned to individual's alpha frequency, or other spatiotemporal dynamics, to enhance treatment efficacy (Jin and Phillips, 2014; Leuchter et al., 2015). Moreover, the combination of TMS-EEG with other neuroimaging modalities including DTI (e.g., Voineskos et al., 2010), fMRI (e.g., Shafi et al., 2015; Farzan et al., 2016), and genetic studies (e.g., Lett et al., 2016) can lead to identification of causal mechanisms that link genetics, brain structure, brain function, and behavior in health and disease. This enhanced understanding of the neuropsychiatric pathophysiology may lead to newer and better treatments.

Finally, combining TMS-EEG with brain computer interface (BCI) provides the possibility to perform closed-loop state-dependant brain stimulation, which has significant potential for neurorehabilitation and neurotherapeutics (refer to Zrenner et al., 2016 for a recent commentary). Such closed-loop systems would permit translation of an intended behavior into action or enable state-dependant neurostimulation therapies. For example, EEG spectral features could detect intention for behavior (e.g., movement) and trigger TMS administration to initiate behavior (e.g., movement execution), such as by TMS-induced activation of networks, that would in turn operate a prosthetic device *via* a BCI interface. Another application, of interest for epilepsy, uses online monitoring of epileptiform EEG activity coupled with closed-loop adjustment of TMS parameters to trigger or prevent seizures for diagnostic and therapeutic purposes (Rotenberg, 2010). The main challenge in closed-loop state-dependent brain stimulation remains the successful online detection of the relevant EEG feature(s), which can be biased by the TMS-related artifact (Walter et al., 2012). In this regard, interpolation of TMS-related after effects was shown to lead to overestimation of decoding performance. This issue was remedied by employing the Burg algorithm (Walter et al., 2012). The future of BCI coupled with TMS-EEG relies in part on advancement and validation of methods for online correction of the TMS-related artifacts, with the goal of optimizing reliable detection of the EEG features.

## Conclusion

The combination of TMS with EEG offers a powerful tool for brain research. TMS-EEG experiments conducted to date illustrate the important discoveries that TMS-EEG has contributed in basic and cognitive neuroscience and clinical research. Combining TMS-EEG with neuroimaging techniques and genetic studies, coupled with equivalent markers from animal and *in-vitro* studies, is increasing the potential of this technique in revealing causal mechanisms governing the gene-brain-behavior relationship. To realize the full potential of TMS-EEG and integrate outcomes across studies, the TMS-EEG study design, and data acquisition and processing should be utilized with special care. Moreover, efforts should be made to report and control the factors that impact the TMS-EEG outcomes and data interpretation. The future of TMS-EEG would benefit from advancement of EEG and TMS equipment, and the standardization and validation of EEG signal processing techniques. As computing power increases, faster adaptive algorithms capable of online detection and removal of EEG artifacts will enable the practical utility of TMS-EEG in closed-loop applications, with significant new potentials in neurotherapeutics and precision medicine.

## Author contributions

FF wrote the manuscript. All authors contributed to the conception of the paper, revising it critically, and approving the final version. All authors are accountable for all aspects of the work.

### Conflict of interest statement

AP serves on the scientific advisory boards for Nexstim, Neuronix, Starlab, Neuroelectrics, Axilum Robotics, Magstim, Neosync, and Novavision; and is listed as an inventor on several issued and pending patents on the real-time integration of transcranial magnetic stimulation (TMS) with electroencephalography (EEG) and magnetic resonance imaging (MRI). The other authors declare that the research was conducted in the absence of any commercial or financial relationships that could be construed as a potential conflict of interest.
